# The influence of electrocardiogram (ECG) filters on the heights of R and T waves in children

**DOI:** 10.1038/s41598-022-17680-4

**Published:** 2022-08-02

**Authors:** Jun Hirokawa, Takashi Hitosugi, Yoichiro Miki, Masanori Tsukamoto, Fumiyasu Yamasaki, Yoshifumi Kawakubo, Takeshi Yokoyama

**Affiliations:** 1 Department of Anesthesia, Miyazaki Dental Welfare Center, Miyazaki City Dental Association, Miyazaki, Miyazaki Japan; 2 Section of Dental Anesthesiology, Division of Maxillofacial Diagnostic & Surgical Sciences, Faculty of Dental Science, Kyushu University, Fukuoka, Fukuoka Japan; 3 School of Interdisciplinary Science and Innovation, Faculty of Arts and Science, Kyushu University, Nishi, Fukuoka Japan; 4 Department of Dental Anesthesiology, Kyushu University Hospital, Fukuoka, Fukuoka Japan; 5 Department of Clinical Laboratory, Kochi Medical School, Nankoku, Japan; 6 Department of Clinical Engineering, School of Medical Sciences, Jikei University of Health Care Sciences, Osaka, Osaka Japan

**Keywords:** Cardiology, Paediatric research, Software

## Abstract

Anesthesiologists often compare intraoperative and preoperative electrocardiogram (ECG) waveforms in patients undergoing general anesthesia. In addition, many intraoperative ECG monitors have filters for removing electrocautery noise. In pediatric anesthesiology practice, we often note the appearance of elevated T waves—specifically, an increase in their height—with the use of such filters, even though no actual clinical change has occurred, which possibly leads to misdiagnosis. We investigated changes in R and T wave heights and in the T/R ratio according to the use of the strong (S) versus the diagnostic (D) filtering mode during pediatric anesthesiology. Primary outcomes were the dependence of the heights of the R and T waves on the filter mode and the correlation between rates of change in the R- and T-wave heights and heart rate (HR). In the S mode, the height of the R wave was lower (*p* = 0.013, η^2^ = 0.28) and the T/R ratio was higher than the corresponding values in the D mode (χ^2^ = 20.46, *p* < 0.001). The T/R ratios were also higher in the S mode than in the D mode, and when the D mode was changed to the S mode during tachycardia, there was a strong correlation between the rate of reduction in the R wave and HR (r = 0. 573, *p* = 0.041). Significant differences in the heights of the R wave and in the T/R ratio occur when using different intraoperative ECG filtering modes. Specifically, in S mode, a greater relative increase in T wave height may occur due to a significant decrease in R wave height. To avoid spurious diagnoses, anesthesiologists should be familiar with these potentially purely filter-driven changes whenever ECG is intraoperatively monitored.

## Introduction

Generally, electrocardiograms (ECGs) are included in biomonitoring equipment. ECGs are used for cardiac monitoring in many clinical situations^1^. Anesthesiologists intraoperatively observe the ECG waveform of patients under general anesthesia and compared it to the preoperative waveform. They do not diagnose cardiovascular disease by recording a 12-lead ECG but rather focus on the changes that occur continuously and over time, observing a small number of lead ECGs. Changes in the waveform may indicate certain cardiovascular events, such as myocardial ischemia or electrolyte abnormalities, which require immediate examination, diagnosis, and treatment by the anesthesiologist. T waves are often elevated in children, and blood may be drawn for suspected abnormalities. Several types of ECG filters are available to remove noise from electrocautery^2^^.^ Because electrocautery is often used in the operating room, it is common to apply a filter system to clarify the waveform during surgery and anesthesia.

First, the filtering functions that cut high frequencies can affect the original waveform^3,4^. However, in children, the R wave of the ECG contains high frequencies; therefore, compared to other waves, R waves may be particularly susceptible to low-pass filters because of the steepness of the waveform. In children, the R wave width is narrower than that in adults and contains many high frequencies^5^. If the amplitude of the R wave is greatly reduced, the T wave then appears to be relatively increased when assessed by anesthesiologists and clinicians in the operating room. As a result, these physicians may misdiagnose patients due to relative changes in ECG waveform height^6–8^. Many anesthesiologists are not aware that filters change the way waveforms appear. We believe that the significance of this study is to alert the public to the possibility that the filtering function, which was originally applied to prevent misdiagnosis, may conversely lead to erroneous judgments and incorrect treatment.

Therefore, we investigated the effect of an electrocardiographic filtering system on R- and T-wave heights in children undergoing dental procedures under general anesthesia.

## Materials and methods

### Ethics approval

The research was conducted ethically in accordance with the World Medical Association Declaration of Helsinki. The enrolled subjects provided written informed consent, and the study protocol was approved by the Research Ethics Committee of the Hospital (Approval No.: 29-416).

### Study design and research subjects

The subjects of this study were pediatric patients undergoing dental treatment under general anesthesia with an American Society of Anesthesiologists physical status (ASA-PS) of 1 or 2 who were under 6 years old. Informed consent was obtained from the subjects and their guardians, and the consent forms were collected from the guardians. A blood test, electrocardiography, and a chest X-ray were performed as a preoperative examination (to detect hidden problems). Patients with congenital or acquired heart disease, including heart malformation, angina, bundle branch blocks, and notable baseline ST elevation due to “early repolarization pattern” ECGs, were excluded. The purpose of this study was to examine the possibility that the original waveform would be impaired by noise cancellation filters commonly used in intraoperative ECG monitoring. Therefore, no comparative study of individual patient backgrounds was conducted.

### Anesthetic management

No premedication was given. Routine monitoring was applied, including electrocardiography, noninvasive blood pressure, pulse oximetry, and end-tidal carbon dioxide. Anesthesia was induced with inhalation of 8% sevoflurane in oxygen. After peripheral intravenous catheterization, nasotracheal intubation was facilitated with intravenous administration of rocuronium 0.6 mg/kg and atropine 0.02 mg/kg, and mechanical ventilation was used for intraoperative respiratory management. Anesthesia was maintained with inhalation of anesthetic agents, including sevoflurane (2.5~3%) or isoflurane (1~1.5%), in an oxygen/air (55%) mixture and an intermittent bolus of fentanyl.

### ECG monitoring and recording

**Figure 1:**
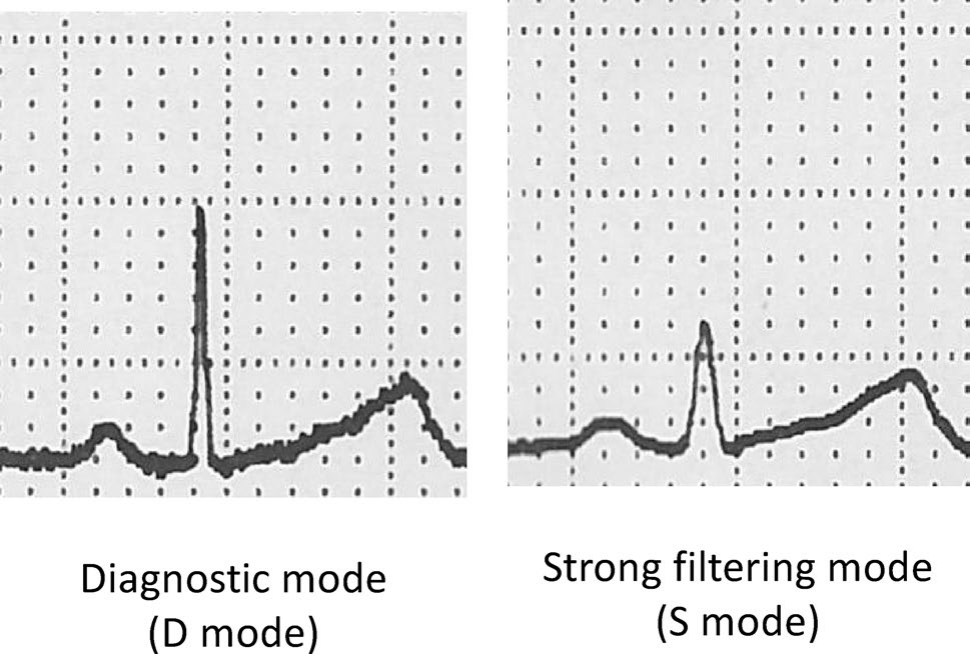
Changes in R- and T-wave heights depending on the different filter modes.

This study investigated changes in the R- and T-wave height and T/R ratio depending on the different filter modes (Fig. 1). These measurements were compared with the strong filtering mode (S mode) or the diagnostic mode (D mode) in BSM-9100 (Nihon Kohden, Tokyo, Japan), which is a biological monitoring tool, and the diagnostic mode in ECG-2550 (Nihon Kohden, Tokyo, Japan) used in the preoperative examination. Each filter mode has different lower/higher limits of cutoff frequencies. The frequency bandwidth of signals in S mode is from 1 to 17 Hz, that in D mode is from 0.05 to 150 Hz and that in the diagnostic mode with ECG-2550 is also from 0.05 Hz to 150 Hz. In addition, we found a correlation between the filter mode of ECG and the height of R and T waves. Limb-lead ECG waveforms were monitored with BSM-9100 and ECG-2550. ECG electrodes were attached to the right forearm, left forearm, and left leg. A ground electrode was attached to the right leg. S mode and D mode were investigated for each patient in this study. The algorithm used for feature extraction was EC-1. After induction of general anesthesia for one hour, we recorded lead II of the ECG in those modes, measured 5 consecutive wave heights, and evaluated the average value. In that same period, heart rate (HR) was also recorded.

### Primary outcomes

The primary outcome of this study was the change in the heights of the R wave and T wave by each filter mode.

Secondary outcomes were the correlations between rates of change in the heights of the R and T waves and HR.

### Statistical analyses

The programming language R (version 4.0.2; The Comprehensive R Archive Network, USA) was used for statistical analysis. The difference was compared between the three filtering modes of the “S mode”, the "D mode", and "control" for each height of the R and T waves and the T/R ratio. The data were subjected to the Shapiro‒Wilk test and Bartlett's test to determine whether they were sampled from a population with a normal distribution and homoscedasticity. It was revealed that for all three groups of R and T wave heights, the T/R ratio had a normal distribution, and R and T were homoscedastic. A paired one-way analysis of variance (ANOVA) was applied to the heights of R and T, which were normally distributed and homoscedastic, and a Friedman test was applied to the T/R ratio. Next, we investigated which filtering modes differed in R by Tukey's HSD test and in the T/R ratio by the Wilcoxon signed-rank test. Pearson correlation coefficients were calculated to assess the linear relationship between HR and the rate of change of the three variables due to switching from D mode to S mode.

### Informed consent

Informed consent was given to the subjects and their guardians, and consent forms were obtained from the guardians.

## Results

### Patient

Thirteen patients participated in this study (Table 1). All patients underwent dental treatment due to multiple dental caries, and they all were evaluated as ASA-PS 1. No adverse events occurred during the study.

**Table 1:** Background: characteristics of the patients.

Number of subjects	13
Female/male	7/6
Age (month)	62.9 ± 13.0
Body height (cm)	106.1 ± 7.7
Body weight (kg)	17.7 ± 3.2
Inhalation anaesthetics (sevoflurane/isoflurane)	9/4

### Measurements

**Figure 2:**
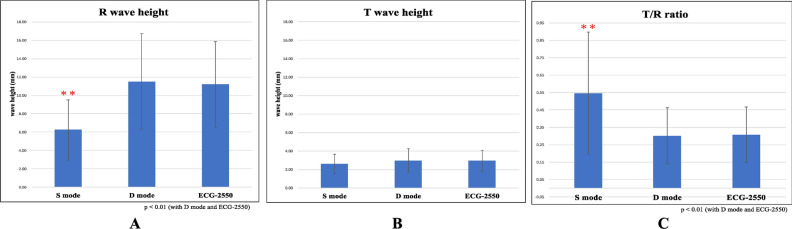
(**A**) Measurements of the R wave heights, (**B**) measurements of the T wave heights, (**C**) measurements of the T/R ratios. In the S mode, the R wave height was lower and the T/R ratio was significantly higher than those in the other modes. There was no significant difference in T wave height.

The heights of the R and T waves and T/R ratios are shown in Table 2 and Fig. 2. The R wave height was found to be significantly lower in S mode than in D mode in BSM-9100 (Nihon Kohden, Tokyo, Japan) and was significantly lower than that in the control in ECG-2550 (Nihon Kohden, Tokyo, Japan) (Fig. 2A). A one-way analysis of variance (ANOVA) revealed significant differences between these three filtering modes (*F*(2, 36) = 5.713, *p* = 0.007, *η*^2^ = 0.24). Multiple comparisons were performed by the Tukey method, and significant differences were found between S and D modes (*p* = 0.013, *η*^2^ = 0.28) and between S mode and the control (*p* = 0.020, *η*^2^ = 0.29), and no significant difference was found between D mode and the control (*p* = 0.98, *η*^2^ = 0.001).

**Table 2:** The measurements of height of R and T wave and T/R ratios.

	S mode	D mode	ECG-2550
Height of R wave (mm)	6.24 ± 3.29	11.51 ± 5.20	11.19 ± 4.66
Height of T wave (mm)	2.61 ± 1.05	2.95 ± 1.28	2.92 ± 1.13
T/R ratio	0.55 ± 0.35	0.30 ± 0.16	0.31 ± 0.16

No apparent difference was found in the T wave height (Fig. 2B), and a one-way ANOVA revealed no significant difference between these three filtering modes (*F*(2, 36) = 0.353, *p* = 0.705, *η*^2^ = 0.019).

Since a clear difference was seen in the T/R ratio (Fig. 2C), a Friedman test was performed, and the results showed that there were statistically significant differences in the T/R ratio (*χ*^2^ = 20.46, *p* < 0.001). A set of Wilcoxon signed-rank tests revealed that there were statistically significant differences between S mode and D mode (*Z* = 3.67, *p* < 0.001) and between S mode and the control (*Z* = 3.67, *p* < 0.001), and there was no significant difference between D mode and the control (*Z* = 1.60, *p* = 0.110).

**Figure 3:**
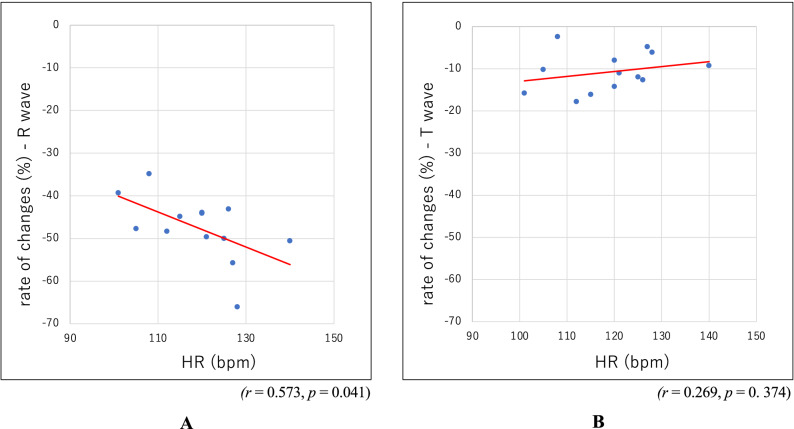
(**A**) Rate of changes in the R waves. The decreasing rate of change in the R wave height from the D mode to the S mode was significantly correlated with the HR. (**B**) Rate of changes in the T waves.

Pearson correlation coefficients were calculated to assess the linear relationship between HR and the rate of change in these variables due to switching from D mode to S mode. A significant positive correlation was found in the rate of change in the R wave height (Fig. 3A, r = 0.573, *p* = 0.041), and no significant correlations were found (Fig. 3B, r = 0.269, *p* = 0. 374).

## Discussion

Electrocardiography is noninvasive cardiac-monitoring procedure that is typically performed on patients undergoing surgery. Because intraoperative noise such as scalpel noise distorts the waveform, many electrocardiographs are equipped with filtering functions that are often used during surgery^2^. There are also several modes of filtering, and their frequency bands vary among electrocardiographs (Table 3). However, we could find no reports examining the effect of filters on monitoring during anesthesia. Consequently, this study is probably the first report on the clinical use of filters during anesthesia.

**Table 3:** ECG devices.

Model	GE	Philips		NIHON KODEN	FUKUDA DENSHI	FUKUDA COLIN
	B650(PDM)	IntelliVue (M8004A MP50/M8003A MP40)		BSM-6301/6501/6701	DS-7000	Accumil V7000
Number of filters	4	3		3	2	4
Filter type (HZ)	D: 0.05~100 M: 0.05~40 Mo: 0.05~25 Max: 5~25	Adult	Pediatric/Newborn	D: 0.05~150 M: 0.3~40 S: 1.0~18	M: 0.5~40 E: 1~20	D: 0.05~150 M: 0.5~40 EK: 1~20 ST: 0.05~40
		D: 0.05~150 Mo: 0.5~40 F: 0.5~20	D: 0.5~150 M: 0.5~150 F: 0.5~20			

*D* Diagnostic mode, *M* Monitor mode, *Mo* Moderate mode, *Max* Maximum mode, *F* Filter mode, *S* Strong monitor, *EK* Electric knife mode, *ST* ST diagnostic mode.

ST measurement requires from 0.05 Hz. From 0.5 Hz, low frequency components are excluded. In addition, it is set uniformly based on the R wave, and accurate ST diagnosis cannot be performed.

GE: ST analysis is possible in each mode. Philips: When making an ST diagnosis, be sure to specify the filter in the diagnostic mode in the instruction manual. (For other filters, the ST of the ECG waveform may look different from the ST of the ST waveform of the same waveform.

Since the ECG waveform is a composite of sinusoidal waves with multiple frequencies^9^, frequency components outside the range of each filtering mode will be cut, potentially distorting the ECG waveform. The R wave is composed of a fundamental wave, a repetitive frequency, harmonics, and integer multiples of that frequency. The heart rate can be regarded as the fundamental frequency among the many frequency components that make up the R wave. As the heart rate (the fundamental frequency) increases, the wave components that make up the R wave shift toward higher frequencies and are more easily removed by the low-pass filter (Fig. 1). In fact, the American Heart Association/American College of Cardiology/Heart Rhythm Society (AHA/ACC/HRS) scientific statement recommends a cutoff of 0.05 Hz for low frequencies and at least 150 Hz for high frequencies in a standard 12-lead ECG routine filter, and this frequency range corresponds to the "D mode" and "control" in this study^10^. The R wave is composed of many high-frequency components, possibly due to the influence of the low-pass filter^5^. Similarly, the possibility of distorting the ECG waveforms in adults due to low pass has been reported^3,4^. The R wave in children was steeper than that in adults; therefore, children may be particularly susceptible.

In the present study, all (100%) of the patients had R wave impairment and an increased T/R ratio. There was little change in the T wave, the R wave height was reduced by approximately 50%, and the T/R ratio increased by more than 70% (Fig. 2). A "relatively high T wave" was significantly observed in the strong filtering mode. There was also a direct correlation between the HR impairment ratio and R wave height (Fig. 3A). These correlations tended to be more pronounced in children with faster heart rates than in adults (data not shown), suggesting that the R wave height is particularly sensitive to the filter mode. This may result in an increase in "relatively high T waves". We found no studies or reports of similar distortion of the ECG waveform occurring with devices from other manufacturers. However, past reports have indicated that changes in the appearance of the ECG (waveform impairment) or possible misdiagnosis may occur depending on the frequency characteristics (cut bandwidth) of the filtering mode.

The present results suggest that distortion of ECG waveform changes may occur not only in children but also in tachycardic adults. Therefore, future studies should also examine the influence on adults and compare it with other companies’ products.

Many clinicians, including anesthesiologists, focus on the relative height of each wave rather than the absolute height of these waves when evaluating ECGs during surgery. This study showed no significant difference in the height of the T wave in each filtering mode. However, it was suggested that a significant decrease in the height of the R wave might increase the appearance of relatively high T waves. These correlations also tend to be more pronounced in children with faster heart rates than in adults. Under these circumstances, clinicians could easily misinterpret an increase in T wave height when there is actually no change in the T wave. As a result, ischemia, electrolyte imbalance, and hyperkalemia may be suspected, and clinicians may make incorrect diagnoses or perform unnecessary blood tests.

## Data Availability

The datasets used during the analysis of the current study are available from the corresponding author upon reasonable request.

## Abbreviations

ECG: Electrocardiogram
ASA: American Society of Anesthesiologists
ACC: American College of Cardiology
HRS: Heart Rhythm Society
S mode: Strong filtering mode
D mode: Diagnostic mode
